# Genetic diversity of the breeding collection of tomato varieties in Kazakhstan assessed using SSR, SCAR and CAPS markers

**DOI:** 10.7717/peerj.15683

**Published:** 2023-07-17

**Authors:** Alexandr Pozharskiy, Valeriya Kostyukova, Marina Khusnitdinova, Kamila Adilbayeva, Gulnaz Nizamdinova, Anastasiya Kapytina, Nazym Kerimbek, Aisha Taskuzhina, Mariya Kolchenko, Aisha Abdrakhmanova, Nina Kisselyova, Ruslan Kalendar, Dilyara Gritsenko

**Affiliations:** 1 Laboratory of Molecular Biology, Institute of Plant Biology and Biotechnology, Almaty, Kazakhstan; 2 Department of Molecular Biology and Genetics, Al Farabi Kazakh National University, Almaty, Kazakhstan; 3 Fruit and Vegetable Research Institute, Almaty, Kazakhstan; 4 Helsinki Institute of Life Science HiLIFE, University of Helsinki, Helsinki, Finland; 5 National Laboratory Astana, Nazarbayev University, Astana, Kazakhstan

**Keywords:** *Solanum lycopersicum*, PCR, Genetic markers, Disease resistance

## Abstract

Tomato is one of the most prominent crops in global horticulture and an important vegetable crop in Kazakhstan. The lack of data on the genetic background of local varieties limits the development of tomato breeding in the country. This study aimed to perform an initial evaluation of the breeding collection of tomato varieties from the point of view of their genetic structure and pathogen resistance using a set of PCR based molecular markers, including 13 SSR markers for genetic structure analysis, and 14 SCAR and CAPS markers associated with resistance to five pathogens: three viruses, fungus *Fusarium oxysporum,* and oomycete *P hytophthora infestans*. Nine SSR markers were with a PIC value varying from 0.0562 (low information content) to 0.629 (high information content). A weak genetic structure was revealed in the samples of varieties including local cultivars and, predominantly, varieties from Russia and other ex-USSR countries. The local varieties were closely related to several groups of cultivars of Russian origin. Screening for a set of resistance markers revealed the common occurrence of the resistance locus *I* against *Fusarium oxysporum* and only the occasional presence of resistance alleles of other markers. No markers of resistance to the three considered viruses were revealed in local tomato varieties. Only two local cultivars had markers of resistance to *P. infestans,* and only the ‘Meruert’ cultivar had a combination of resistance markers against* P. infestans* and* F. oxysporum.* The obtained results have demonstrated the need for further studies of local tomato varieties with a wider range of molecular markers and source germplasm to lay a foundation for the development of tomato breeding in Kazakhstan.

## Introduction

Tomato (*Solanum lycopersicum* L.) is a representative plant species of the Solanaceae family, which includes a number of important vegetable and technical crops. Tomato is one of the most popular vegetable crops all over the world, as well as the closely related species, potato (*Solanum tuberosum* L.) (Camargo Filho & Camargo, 2017).

Tomatoes comprise an important part of overall vegetable production in Kazakhstan, with 788,760 tons harvested from 30.2 thousand hectares in 2022. Tomato production has been developed in the country extensively rather than intensively; the growing area has doubled, but the yield per hectare volume has stagnated in the last 30 years (Food Agriculture Organization of the United Nations, 2021). Among the tomato varieties approved for cultivation in the country, foreign cultivars prevail with a significant share of varieties from Russia and other ex-USSR countries (The Ministry of Agriculture of the Republic of Kazakhstan, 2009). Such a dependence on imported planting material poses various risks for food security, the most concerning of which is the possible importation of dangerous pests (Chalam et al., 2021), weeds (Wilson et al., 2016), and pathogens (Elmer, 2001; Rodoni, 2009). Thus, it is important for the domestic market of agricultural crops to adopt a wider use of old and newly obtained varieties that are bred locally, and it should be associated with comprehensive plant epidemiological controls. To confront potentially deleterious plant pathogens, it is not only necessary to detect and eradicate infected plants in a timely manner, but also to increase the resistance potential of cultivated crops against disease by breeding and selecting varieties with genetic factors of resistance. Modern practices require the extensive utilization of molecular methods to solve both these problems. Molecular markers associated with disease resistance in plants play a crucial role in modern breeding programs since their use in marker-assisted selection (MAS) helps to significantly reduce the time and labor required for developing new resistant varieties (Collard & Mackill, 2008; Miedaner, 2016). Such an approach utilizes molecular markers with known linkage with the target traits to lead selection without the need for direct control of the phenotype, *e.g.*, in the early developmental stages; the practices of MAS are widely utilized in tomato breeding for resistance to pathogens (Foolad & Panthee, 2012). However, in Kazakhstan, the implementation of such advanced breeding practices for tomato is limited by relatively low economic and scientific interests. To date, no systematic efforts have been made to lay the molecular genetic basis for selection programs for tomato crops. In contrast, the molecular genetics of wheat, the crop playing a prominent role in both the country’s domestic food marker and international trade, has received significant research attention for years (Kokhmetova et al., 2017; Anuarbek, Abugalieva & Turuspekov, 2018).

The objective of this work was to investigate the genetic structure of the collection of tomato varieties deposited in the Fruit and Vegetable Research Institute (Almaty, Kazakhstan). The collection included established local cultivars along with varieties from abroad, predominantly from Russia and other ex-USSR countries. Most of them have not been included in the state register of crop varieties recommended for use (The Ministry of Agriculture of the Republic of Kazakhstan, 2009) and thus require extensive investigations of such factors as their genetic compositions, immunity, and physiological features under local growth conditions. Along with previously published data on the genetic markers of resistance against three common viruses (Pozharskiy et al., 2022), this work presents the results of the first molecular genetic study of tomato varieties in Kazakhstan. A set of simple sequence repeats (SSRs), sequence characterized amplified region (SCARs), and cleavage amplified polymorphic sequences (CAPS) markers was used to evaluate the relations between selected cultivars and identify varieties bearing known loci of resistance to common tomato pathogens: oomycete *Phytophthora infestans,* fungus *Fusarium oxysporum,* tomato mosaic virus (ToMV), tomato spotted wilt virus (TSWV), and tomato yellow curly leaf virus (TYLCV). Except for *F. oxysporum,* these pathogens have been included in the list of quarantine objects, invasive species, and dangerous organisms by the Ministry of Agriculture of the Republic of Kazakhstan (The Ministry of Agriculture of the Republic of Kazakhstan, 2015). Three viruses, ToMV, TSMV, and TYLCV, are among the most dangerous tomato pathogens causing significant damage, potentially as much as the total yield loss (Broadbent, 1976; Pico’, Jo Diez & Nuez, 1996; Roselló, Díez & Nuez, 1996). The broad specificity of these viruses to diverse host plant species (Ying & Davis, 2000; Parrella et al., 2003; Hancinský et al., 2020) expands the potential risks of virus propagation beyond tomato culture and makes disease control more challenging. Although, because of the lack of systematic molecular studies of tomato viruses, the presence of these viruses has not been detected to date in Kazakhstan, they are considered potentially threatening quarantine objects, as mentioned above. Previously, we tested a selection of tomato varieties using a set of SCAR and CAPS markers associated with resistance to the three mentioned viruses (Pozharskiy et al., 2022): PrRuG86-151, associated with resistance locus *Tm-2* against ToMV (Lanfermeijer, Warmink & Hille, 2005; markers NCSw-003, NCSw-005, NCSw-011, NCSw-012 (Panthee & Ibrahem, 2013), and Sw5-2 (Dianese et al., 2010), associated with resistant locus *Sw-5* against TSWV; markers Ty2-UpInDel, Ty3-InDel, Ty3-SNP9, and Ty3-SNP17, associated with resistance locy *Ty-2* and *Ty-3* against TYLCV (Kim et al., 2020). Here, we tested these markers on additional tomato samples from the local collection.

Oomycetes of the *Phytophthora* genus are among the most destructive plant pathogens, and *P. infestans* is the most threatening pathogen of potato and tomato, potentially causing total yield losses at the regional level (Legard, 1995; Judelson & Blanco, 2005; Ismailova et al., 2017; Nowicki et al., 2012; Jung et al., 2015). In Kazakhstan, *P. infestans* is among the most common tomato infections caused by fungus-like organisms (Ismailova et al., 2017). Due to the high genetic variability of this pathogen, the known resistance loci in tomato have only limited protective effect specific to particular *Phytophthora* isolates (Nowicki et al., 2012). The CAPS markers used here, TG328 and Ph3-gsm, are linked with the *Ph-3* resistance locus (Robbins et al., 2010; Wang et al., 2016), which confers partial resistance to a range of Phytophthora isolates and is widely used in breeding practices (Jung et al., 2015).

*Fusarium oxysporum* is a soil fungus capable of causing an opportunistic infection in a wide range of susceptible plants, including tomato; the hyphae of the fungus can penetrate the roots and colonize xylem vessels, causing vascular wilt (Pietro et al., 2003). The sub-species *F. oxysporum* f.sp. *lycopersici* (*Fol*) is the main causative agent of vascular wilt in tomato; three races are known, and for each of them the corresponding genetic factors of resistance have been described (Chitwood-Brown et al., 2021). The presence of multiple *F. oxysporum* f.sp. *lycopersici isolates* has been detected in Kazakhstan (Sagitov, El-Habbaa & El-Fiki, 2010). Here, we tested our collection using dominant SCAR markers *At2* and *Z1063*, associated with resistance loci *I* and *I-2 (Arens et al., 2010),* conferring resistance to races *Fol-1* and *Fol-2* (Chitwood-Brown et al., 2021).

This work aimed to fill the existing knowledge gap in the genetic basis of tomato breeding and Kazakhstan, to test the applicability of known genetic markers to local tomato varieties, and to identify genotypes bearing resistance markers against several important pathogens. As no studies of the genetic diversity of tomato have been lead to date in Kazakhstan, the obtained results will provide novel data on the state of tomato breeding in the country and help lay a basis for an initial inventory of tomato plant materials to be used both in agriculture and in breeding programs in Kazakhstan.

## Materials and Methods

A selection of tomato varieties was obtained from the collection of the Fruit and Vegetable Research Institute (FVRI; Almaty, Kazakhstan) (Table 1). Seed materials were grown and DNA was isolated as previously described in Pozharskiy et al. (2022).

**Table 1:** List of studied tomato varieties.

Sample ID	Variety name		Country of origin	Included to the State Register	Sample ID	Variety name		Country of origin	Included to the State Register
T001	Choportula^*^		Georgia		T290	Gribnoye Lukoshko		Russia	
T003	Zagadka Prirody^*^	[Enigma of Nature]*^***^	Russia		T292	Sladkoyezhka	[Sweet-tooth]	Kazakhstan	
T005	Idillia^*^	[Idyll]	Russia		T296	Super Exotic		Russia	
T007	Yablochnyi^*^	[Apple-like]	Uzbekistan		T314	Ranniy310^*^	[Early 310]	Belarus	
T008	Shalun^*^	[Varmint]	Russia		T316	Yarkiy Rumyanets^*^	[Bright Blush]	Russia-Kazakhstan^**^	
T010	Uragan^*^	[Hurricane]	Serbia		T317	N7952691322^*^		Russia	
T012	Semka^*^	[Seed]	Russia		T319	Malinovyi Slon^*^	[Crimson Elephant]	Russia	
T013	Pavlina^*^		Russia		T320	Palmira^*^		Russia	
T016	Pozhar^*^	[Fire]	Belarus		T322	Lambrusko^*^		Russia	
T018	Rassvet^*^	[Sunrise]	Kazakhstan	+	T325	Principe Borghese		Italy	
T019	Denar^*^		Netherlands		T328	Tolstushka^*^	[Fatty]	Russia	
T020	Hybrid16155		Kazakhstan		T330	Local with Carrot- Leaf		Kazakhstan	
T022	Korolek	[Kinglet]	Russia		T333	Rassvet362^*^	[Sunrise 362]	Russia	+
T024	Spiridon^*^		Russia		T335	Anait		Armenia	
T025	Venera^*^	[Venus]	Kazakhstan		T336	33 Bogatyrya	[33 Heroes]	Russia	
T026	Grapefruit		Russia		T338	Yablochnyi^*^	[Apple-like]	Kazakhstan	
T053	Yusupovskiy		Uzbekistan		T340	Magnat		Russia	
T078	Lipen^*^		Ukraine		T341	Malvina		Russia	
T114	Zhiraf^*^	[Giraffe]	Russia		T343	Tuzlovets		Russia	
T122	Dama^*^	[Dame]	Ukraine		T444	Krasnaya Presnya	[Red Presnya]	Russia	
T150	Heart-likeRed		Kazakhstan		T466	Pobeditel	[Winner]	Russia	
T170	Zhirik		Russia		T479	Malets	[Small Boy]	Russia	
T185	Malika^*^		Russia		T496	Nicola^*^		Russia	
T187	Ruzha^*^		Belarus		T512	Russian Delicacy		Russia	
T194	Kozyr^*^	[Trump]	Russia		T539	Gloria		Moldova	
T211	Sunnik^*^		Armenia		T562	Mechta	[Dream]	Kazakhstan	
T217	Costoluto Biorentino^*^		Italy		T595	Meruert		Kazakhstan	+
T221	Monach^*^	[Monk]	Russia		T606	Novichok	[Newcomer]	Russia	+
T235	Pyatnitca	[Friday]	Russia-Kazakhstan^**^		T609	Vostorg	[Delight]	Kazakhstan	+
T237	Barmaley		Russia		T612	Luchezarnyi	[Shiny]	Kazakhstan	+
T247	Kolokola Rossii	[Russian Bells]	Russia		T625	Samaladay		Kazakhstan	+
T257	Ayan		Kazakhstan		T628	Yantarnyi	[Amber]	Kazakhstan	
T262	Orange-Violet		Kazakhstan		T631	Leader		Kazakhstan	+
T266	Lilliput Hybrid F1		Italy		T634	Samaladay^***^		Kazakhstan	+

**Notes.**

* Data on resistance markers against ToMV, TSWV, TYCLV taken from Pozharskiy et al. (2022).

** Local breeding line based on Russian cultivars.

*** Intermediate breeding line.

**** Translations of the Russian names of cultivars.

SSR genotyping was conducted using known markers (Table 2) (Smulders et al., 1997; Areshchenkova & Ganal, 2002). Forward primers labeled with either fluorescein (FAM) or hexachlorofluorescein (HEX) were used for all markers. The polymerase chain reaction (PCR) conditions were set in accordance with the corresponding published protocols. The presence of PCR products was confirmed by electrophoresis in 1% agarose gel with 1x tris-acetate buffer, and then the fragment sizes (alleles) were determined by capillary electrophoresis using a 3500 Genetic Analyzer (Applied Biosystems, Thermo Fisher Scientific, Waltham, MA, USA). PCR samples were 20-fold diluted and combined into groups for multiplex fragment reading. Three groups were defined based on the used primer labels and expected fragment size ranges of the markers, to avoid overlaps between markers and to ensure the independent detection of alleles. The diluted PCR mixes were added to high-purity formamide (1 µl PCR mix, 0.15 µl LIZ(-500) Size Standard (Applied Biosystems, Thermo Fisher Scientific, Waltham, MA, USA), 8.85 µl formamide), denatured at 95 °C for 4 min, cooled on ice for 5 min, and loaded for capillary electrophoresis. Genotypes were determined using GeneMapper software and analyzed using a Bayesian approach implemented in MrBayes (Ronquist et al., 2012) and STRUCTURE (Pritchard, Stephens & Donnelly, 2000) software. R language (R Core Team, 2019) with the additional packages, indicated below, was used for general data handling and visualization. The genotyping data were encoded using an additive pseudo-haploid scheme where each observed allele was represented as a single digit value: 0 for absence, 1 for heterozygous state, and 2 for homozygous state. Minor allele frequency, and expected and observed heterozygosity for each marker were calculated using the ‘adegenet’ R package (Jombart, 2008; Jombart & Ahmed, 2011). The polymorphism information content (PIC) was calculated using the method (Botstein et al., 1980) implemented in the ‘polysat’ R package (Clark & Jasieniuk, 2011).

**Table 2:** Tomato SSR markers used for genotyping.

Marker name	PCR primers	Repeating pattern^*^	Expected allele range	Multiplex group	Source
LE20592	F: 5′- FAM -CTGTTTACTTCAAGAAGGCTG R: 5′-ACTTTAACTTTATTATTGCCACG	(TAT)_15−1_(TGT)_4_	165–172	1	Smulders et al. (1997)
LE21085	F: 5′- FAM -CATTTTATCATTTATTTGTGTCTTG R: 5′-ACAAAAAAAGGTGACGATACA	(TA)_2_(TAT)_9−1_	103–119	1	
LELE25	F: 5′- FAM -TTCTTCCGTATGAGTGAGT R: 5′-CTCTATTACTTATTATTATCG	(TA)_13−1_	222–225	2	
LELEUZIP	F: 5′- HEX -GGTGATAATTTGGGAGGTTAC R: 5′-CGTAACAGGATGTGCTATAGG	(AAG)_6−1_TT	101-105	2	
LEMDDNA	F: 5′- HEX -ATTCAAGGAACTTTTAGCTCC R: 5′-TGCATTAAGGTTCATAAATGA	(TA)_9_	210-226	3	
LEPRP4	F: 5′- HEX -TTCATTTCTTGCAACTACGAT R: 5′-CATACTAGCAACATCAAAGGG	(TAT)_3_(TGT)_5_	108-112	3	
LESODB	F: 5′- FAM -TTATCAATTCATCATTGTGGC R: 5′-AGTAAGGGGTTTAGGGGTAGT	(TTC)_6_	208–212	1	
LEATRACAb	F: 5′- FAM -GTATGTCAAATCTCTCTTGCG R: 5′-ACTCTCCATCGTCTCTTTCAC	(GA)_7_	184–186	2	
LPHSF24	F: 5′- HEX -TTGGATTTACAAGTTCGATGT R: 5′-GCATTTGACTTGATAGCAGTC	(TA)_6_	156–158	1	
LECHSOD	F: 5′- FAM -TTATCAATTCATCATTGTGGC R: 5′-AGGGGTAGTGACAGCATAAAG	(CTT)_6_	196–198	3	
LEMDDNb	F: 5′- FAM -TAAATACAAAAGCAGGAGTCG R: 5′-GAGTTGACAGATCCTTCAATG	(TG)_4_(TA)_4_	278–280	2	
TMS63	F: 5′- HEX -GCAGGTACGCACGCATATAT R: 5′-GCTCCGTCAGGAATTCTCTC	(AT)_4_(GT)_18_(AT)_9_	130–150^**^	2	Areshchenkova & Ganal (2002)
TMS58	F: 5′- HEX -CATTTGTTGTATGGCATCGC R: 5′-CAGTGACCTCTCGCACAAAA	(TA)_15_(TG)_17_	223–226^**^	3	

**Notes.**

* (-1) at the subscript indicates the presence of an imperfect repeat.

** According to Mazzucato et al. (2008); otherwise according to Castellana et al. (2020).

MrBayes was run for 50,000,000 generations with the Dirichlet distribution model for standard data; every 2,000th generation was sampled and used for diagnostics by the average standard deviation of tree probabilities in two parallel runs. The parameters of the run were monitored using built-in MrBayes statistics and Tracer (Rambaut et al., 2018), and the summary tree was generated using a burn-in threshold of 50%. The ‘ggtree’ R package (Yu et al., 2017) was used to visualize the summary tree along with the data mentioned below.

The STRUCTURE analysis was run for expected numbers of clusters *K* from 1 to 10 using the standard admixture model with 50,000 burn-in and 100,000 Markov chain Monte-Carlo (MCMC) iterations. To find the optimal *K,* ten replicates were calculated for each *K* value, and the CLUMPAK web-server (Kopelman et al., 2015) was used to estimate Δ*K* following Evanno’s method (Evanno, Regnaut & Goudet, 2005).

PCR was performed for previously known markers of resistance against pathogens in accordance with published protocols (Table 3). All PCR products were checked using agarose gel electrophoresis. Markers requiring restriction (CAPS) were digested by corresponding enzymes in a 20 µl mix containing 5 µl of the PCR mix, 0.5 µl of enzymes, and 2 µl of the appropriate restriction buffer, according to the manufacturer’s recommendations. Restriction was performed overnight with the regular enzyme or for an hour with the enzymes of the FastDigest™ product series (Thermo Fisher Scientific, Waltham, MA, USA). The results of the restriction were evaluated by electrophoresis in 1.5% agarose gel with 1x tris-acetate buffer. All results of the genotyping by resistance markers were interpreted in accordance with the results reported in the source publications. For 31 specimens, previously published data on ToMV, TSWV, and TYCLV resistance markers were used for comparison (Pozharskiy et al., 2022), as indicated in Table 1.

**Table 3:** Tomato SCAR and CAPS markers associated with resistance to pathogens.

Pathogen	Resistance locus	Linked marker	PCR primers	Restriction enzyme	Source
*Phytophtora infestans*	*Ph-3*	CAPS Ph3.gsm	F: 5′-TAGTATGGTCAAACATATGCAG R: 5′-CTTCAAGTTGCAGAAAGCTATC	FD *Hin* cII	Wang et al. (2016)
		CAPS TG328	F: 5′-GGTGATCTGCTTATAGACTTGGG R: 5′-AAGGTCTAAAGAAGGCTGGTGC	FD *Mva* I (*Bst* NI)^***^	Robbins et al. (2010)
*Fusarium oxysporum*	*I*	SCAR At2	F: 5′-CGAATCTGTATATTACATCCGTCGT R: 5′-GGTGAATACCGATCATAGTCGAG + control (LAT): F: 5′-AGACCACGAGAACGATATTTGC R: 5′-TTCTTGCCTTTTCATATCCAGACA	–	Arens et al. (2010)
	*I2*	SCAR Z1063	F: 5′-ATTTGAAAGCGTGGTATTGC R: 5′-CTTAAACTCACCATTAAATC + control (Rubisco): F: 5′-ATGTCACCACAAACAGAGAC R: 5′-CTCACAAGCAGCAGCTAG	–	
Tomato mosaic virus (ToMV)	*Tm2*	CAPS PrRuG086-151	F: 5′-GAGTTCTTCCGTTCAAATCCTAAGCTT GAGAAG R: 5′-CTACTACACTCACGTTGCTGTGATGCAC	*Ksp* AI (*Hpa* I)^***^	Lanfermeijer, Warmink & Hille (2005)
Tomato spotted wilt virus (TSWV)	*Sw-5*	SCAR NCSw-003	F: 5′-TCTCGTTATCCAATTTCACC R: 5′-GCAATTTTGTTTCTTGGTCT	–	Panthee & Ibrahem (2013)
		SCAR NCSw-012	F: 5′-ATGGTCAACTCGATCAGAAC R: 5′-TTTGGTGAGGATCTGATTTC	–	
		CAPS NCSw-007	F: 5′-GTTGCTAACTCGACTCGTTC R: 5′-TCACTCACGTCCTATTGACA	FD *Hin* fI	
		CAPS NCSw-011	F: 5′-TATCATCCTCATACCCCTTG R: 5′-GGATTTTCTCATCATCTCCA	*Hpy* F3I (*Dde* I)^***^	
		SCAR Sw5-2	F: 5′-AATTAGGTTCTTGAAGCCCATCT R: 5′-TTCCGCATCAGCCAATAGTGT	–	Collard & Mackill (2008)
Tomato yellow curly leaf virus (TYLCV)	*Ty-2*	SCAR Ty2-UpInDel	F: 5′-ACCCCAAAAACATTTCTGAAATCCT R: 5′-TGGCTATTTTGTGAAAATTCTCACT	–	Kim et al. (2020)
	*Ty-3*	CAPS Ty3-InDel/SNP9	F: 5′-CCTATCCTCAGTGTTTCGGTCA R: 5′-GGCGAAAGACTTTGTGTACACA	*Bst* 1107I (*Bst* Z17I) / *Mun* I (*Mfe* I)^***^	
		CAPS Ty3-SNP17	F: 5′-TCTCAGGTGATGCTGAGCAC R: 5′-AGAGAACGAAAACGAAATTTCAAACA	*Rsa* I	

**Notes.**

* Gene ID and genomic positions according *S. lycopersicum* genome assembly SL3.0.

** Marker positions in *S. lycopersicum* genome assembly SL3.0.

*** Isoschizomers used in the work and by the original authors (in parentheses).

FD FastDigest™ restriction enzyme product series (Thermo Fisher Scientific, USA)

PCR conditions for all markers used in the study are shown in File S1.

For all individual PCR reactions, both for SSR and resistance markers, the samples failing to produce a result were re-processed at least twice. If no results were obtained in any replicate, the genotype was reported as missing.

## Results

A total of 68 tomato varieties were used in this study, including 13 cultivars of domestic origin. Most of these varieties represent a pool of tomato genotypes used in ongoing breeding programs. The local cultivars ‘Meruert’, ‘Vostorg’, ‘Luchezarnyi’, and ‘Samaladay’, as well as the Russian cultivars ‘Novichok’ and ‘Rassvet 362’, have also been approved for commercial use in Kazakhstan (The Ministry of Agriculture of the Republic of Kazakhstan, 2009).

According to the results of SSR genotyping, four markers—LEPRP4, LESODB, LECHSOD, and LEMDDNb—were revealed to be monomorphic across all tomato varieties (Table 4). LEPRP4 also had the highest missing genotype rate among all markers (11.76%). Markers LELE25, LELEUZIP, and LECHSOD were amplified in all studied samples. None of the other markers exceeded a missing rate of 7.35%, corresponding to 5 of 68 missing samples. Among the polymorphic markers, LEATRACAb, LPHSF24, and TMS58 had levels of observed heterozygosity that did not significantly differ from the expected values. The LEMDDNA marker had a slightly higher observed heterozygosity (*p-* value 0.0003; significance level 0.001); the other five markers had significantly lower observed values compared to the expected values (*p-* values near zero). Considering the nature of the studied samples, which comprised a heterogeneous set of specimens of different varieties rather than a single population, we did not expect the samples to follow Hardy–Weinberg equilibrium, and thus deviations between the expected and observed levels of heterozygosity were not surprising. Although the volume and heterogeneity of the samples limited any possible genetic inferences of the population, it could be speculated that the LEATRACAb, LPHSF24, and TMS58 markers were neutral with respect to the selection of tomato varieties. Markers LELEUZIP and LEMDDNA were revealed to be the most informative for the genotype discrimination, based on calculated PIC values 0.5328 and 0.629, respectively. Markers LEATRACAb and LPHSF24, in contrast, had low PIC values, 0.0570 and 0.0562, respectively. Five other polymorphic markers had moderate information content, with PIC values varying from 0.2035 (LELE25) to 0.3253 (TMS63).

**Table 4:** Summary of SSR genotyping of 68 tomato varieties.

Marker name	*N*	Detected alleles	Missing genotype rate	MAF	*H*_*e*_	*H*_*o*_	*H*_*e*_vs. *H*_*o*_ (*χ*^2^ test *p-* value)	PIC
LE20592	3	164,167,170	0.0147	0.1045	0.3206	0.0149	0	0.2972
LE21085	2	103,117	0.0441	0.1769	0.2912	0.0154	2.2315×10^−14^	0.2488
LELE25	3	218,220,222	0	0.0735	0.3334	0.2059	6.7279×10^−14^	0.2035
LELEUZIP	4	102,104,105,106	0	0.3088	0.5978	0	0	0.5328
LEMDDNA	5	211,213,219,227,233	0.0147	0.2463	0.6788	0.7164	0.0003	0.6290
LEPRP4	1	201	0.1176	–	–	–	–	–
LESODB	1	207	0.0294	–	–	–	–	–
LEATRACAb	2	184,186	0.0294	0.0303	0.0588	0.0606	0.7995	0.0570
LPHSF24	2	158,164	0.0147	0.0298	0.0579	0.0597	0.8011	0.0562
LECHSOD	1	195	0	–	–	–	–	–
LEMDDNb	1	277	0.0147	–	–	–	–	–
TMS63	4	158,184,188,202	0.0735	0.2222	0.3818	0.0793	0	0.3253
TMS58	3	226,228,230	0.0735	0.1667	0.3287	0.3333	0.8085	0.2916

**Notes.**

*N* number of detected alleles MAF minor allele frequency *H*_*e*_ expected heterozygosity *H*_*o*_ observed heterozygosity PIC polymorphism information content

**Figure 1:**
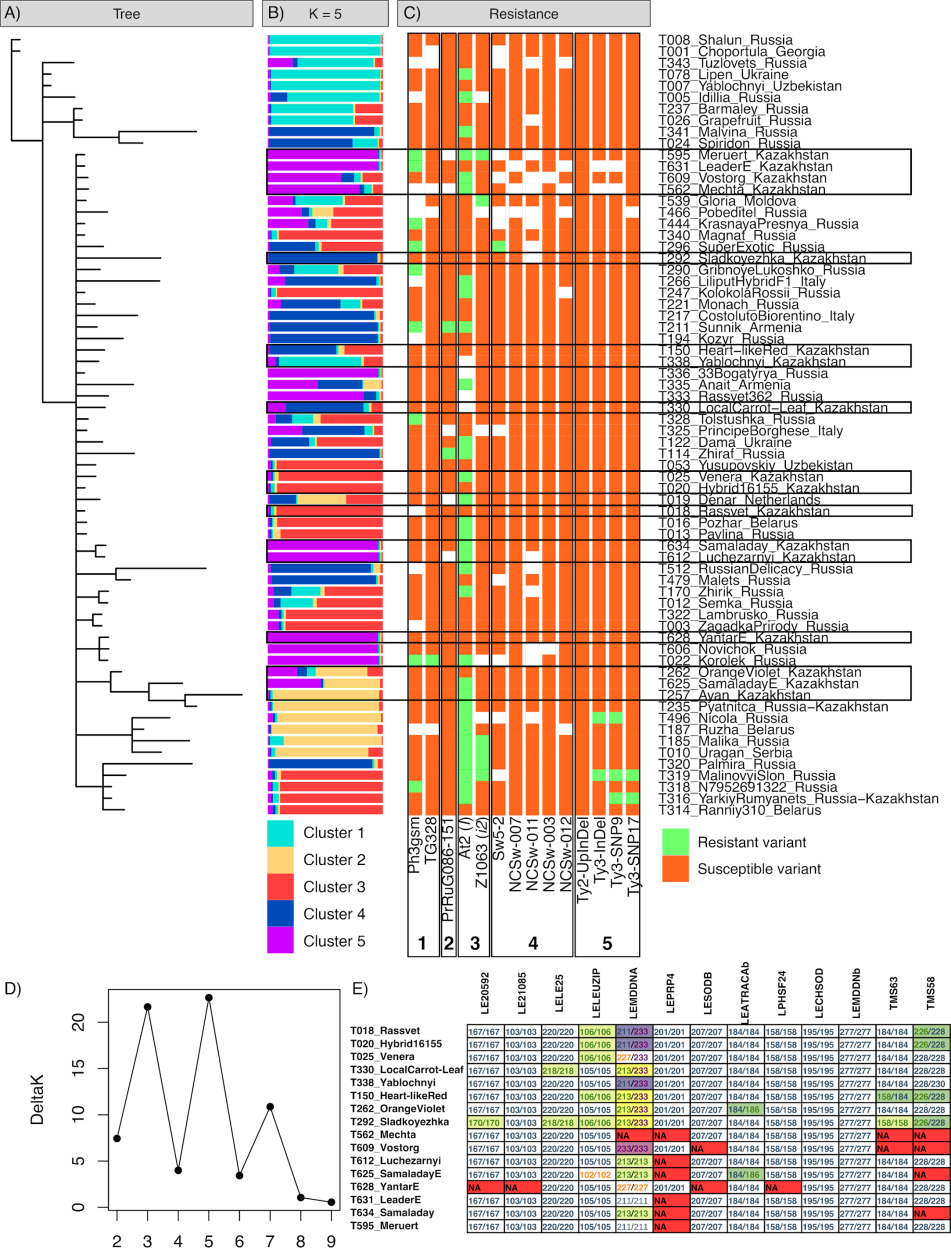
Results of the genotyping of tomato varieties with SSR markers and markers associated with disease resistance. (A) Bayesian tree of varieties based on SSR markers. (B) STRUCTURE plot for five cluster configurations based on SSR markers. (C) tomato genotypes in markers of resistance against *Phytophtora infestans* (1), ToMV (2), *Fusarium oxysporum* (3), TSWV (4), and TYCLV (5). (D) Evanno’s Δ*K* plot indicating the optimal *K.* (E) variations of SSR genotypes in tomato varieties of Kazakhstani origin.

The genetic heterogeneity of the studied samples was revealed by Bayesian cluster analysis (Figs. 1A, 1B). The results obtained using two algorithms implemented in MrBayes and STRUCTURE software were compared to acquire a more detailed picture of the genetic structure of the samples. According to the MrBayes results, most of the studied tomato varieties formed a large subtree with a weak sub-structure. The results obtained with STRUCTURE produced a data partition into five clusters, in accordance with the best Evanno’s ΔK value (Fig. 1D). The first cluster (shown cyan) was the most distinct group representing a compact sub-group at the tree; the highest probabilities were assigned to the ‘Lipen’ (Ukraine), ‘Yablochnyi [Apple-like]’ (Uzbekistan), ‘Choportula’ (Georgia), and ‘Shalun [Varmint]’ (Russia) cultivars, which had identical genotypes. The local variant of the ‘Yablochnyi [Apple-like]’ cultivar was the only variety from Kazakhstan included in this cluster; however, it was located apart from other varieties in the tree and differed from its Uzbekistani relatives in two markers, LE21085 and TMS58. Another distinct cluster (shown in yellow) included two small subclusters in the tree; the typical members of this group were the ‘Ayan’ (Kazakhstan), ‘Ruzha’ (Belarus), ‘Nicola’ (Russia), and ‘Pyatnica [Friday]’ (local breeding line based on Russian cultivar) cultivars. The other three clusters (shown in red, blue, and purple) appeared as a mixed set of subgroups and intermediate genotypes within the main subtree.

Fifteen tomato varieties resulted from breeding efforts established in Kazakhstan. All local varieties yielded a high genetic similarity according to the SSR markers used (Fig. 1E; File S2). Across all 11 polymorphic markers, only three markers demonstrated genotype variations within the local cultivars: LEMDDNA with detected alleles 211, 213, 227, and 233; LELEUZIP with alleles 102, 105, and 106; and TMS58 with alleles 226, 228, and 230. The LELE25, LEATRACAb, and TM63 markers had only two differing genotypes across 15 local varieties, and marker LE20592 had the only differing genotype in the ‘Sladkoyezhka’ cultivar. This cultivar was the most distinct across all local varieties. The ‘Yantar [Amber]’, ‘Leader’, ‘Luchezarnyi [Shiny]’, ‘Meruert’, ‘Vostorg [Delight]’, and ‘Mechta [Dream]’ varieties formed a group of similar genotypes (purple color in Fig. 1B), along with Russian cultivars ‘Novichok [Newcomer]’, ‘Korolek [Kinglet]’, ‘Rassvet 365 [Sunrise 365]’, and ‘33 bogatyrya [33 heroes]’. The breeding line of the ‘Samaladay’ cultivar (specimen T634) also belonged to this group; however, the finally established line for commercial use (specimen T625) differed in the LELEUZIP (genotype 102/102) and LEATRACAb (184/186) markers. The LEPRP4 and TMS58 markers were characterized by a notably high occurrence of missing genotypes in this group. These local varieties were obtained by the breeding programs of the former Research Institute of Potato and Vegetable Breeding (now part of the Fruit and Vegetable Research Institute, Almaty, Kazakhstan) (Kurganskaya & Dzhantasova, 2005). Other local varieties were more diverse, in relation to various foreign cultivars.

The analysis of SCAR and CAPS markers associated with resistance against infections revealed the prevailing presence of resistance loci to fungus *Fusarium oxysporum* and oomycete *Phytophtora infestans,* compared to viruses (Table 5, Figs. 1C; File S3). The most commonly occurring marker was At2, associated with resistance locus *I* against *F. oxysporum*; half of all 64 successfully genotyped samples were positive for resistance. Another resistance marker against *F. oxysporum,* Z1063, associated with *I2* resistance genes, was observed in six specimens, including the local ‘Meruert’ cultivar. Both markers are dominant SCAR markers linked with the corresponding resistance loci introduced to tomatoes from *Solanum pimpinellifolium (Arens et al., 2010).* Two codominant markers, Ph3-gsm and TG328, have been linked with the *Ph-3* locus conferring resistance to *P. infestans* (Robbins et al., 2010; Wang et al., 2016). Two local cultivars, ‘Meruert’ and ‘Leader’, had the resistant allele of Ph3-gsm; the only specimen with the resistant variant of TG328 was the Russian cultivar ‘Korolek [Kinglet]’. Only two cultivars had the resistant allele of marker PrRuG086-151 associated with locus *Tm-2* conferring resistance to ToMV (Lanfermeijer, Warmink & Hille, 2005), Russian cultivar ‘Zhiraf [Giraffe]’ and Armenian ‘Sunnik’, as was previously revealed by Pozharskiy et al. (2022). Almost no markers associated with the resistant locus *Sw-5* against TSWV (Dianese et al., 2010; Kim et al., 2020) were detected, with the exception of marker Sw5-2 in the Russian ‘Super exotic’ variety. For TYCLV, markers associated with resistance loci *Ty-2* and *Ty-3* were tested (Kim et al., 2020). No resistant allele for the marker Ty2-UpInDel was revealed. Three markers associated with the resistant variant of *Ty-3* were previously identified in Russian cultivars (Pozharskiy et al., 2022).

**Table 5:** Summary of the genotyping results of 68 tomato varieties with SCAR and CAPS markers of resistance against infectious diseases.

Pathogen	Marker	Marker type	Susceptible genotypes		Resistant genotypes		Missing data counts
			Fragment sizes^**^	Counts	Fragment sizes^**^	Counts	
*Phytophtora infestans*	Ph3.gsm	CAPS	596 + 501 + 107	48	596 + 291 + 258	9	11
	TG328	CAPS	500	62	260+240	1	5
*Fusarium oxysporum*	At2	SCAR	92	32	130+92	32	4
	Z1063	SCAR	1380	57	1380+940	6	5
Tomato mosaic virus (ToMV)	PrRuG086-151^*^	CAPS	NA^***^	61	NA^***^	2	5
Tomato spotted wilt virus (TSWV)	NCSw-003^*^	SCAR	600	66	680	0	2
	NCSw-012^*^	SCAR	1000	62	–	0	6
	NCSw-007^*^	CAPS	240	65	480	0	3
	NCSw-011^*^	CAPS	600	53	430+200	0	15
	Sw5-2^*^	SCAR	510 or 464	56	574	1	11
Tomato yellow curly leaf virus (TYLCV)	Ty2-UpInDel^*^	SCAR	213	68	120	0	0
	Ty3-InDel^*^	CAPS	669	64	353+325	2	2
	Ty3-SNP9^*^	CAPS	555+114	63	678	3	2
	Ty3-SNP17^*^	CAPS	562 + 148 + 52 + 51	65	497 + 148 + 65 + 52 + 51	2	1

**Notes.**

* Including data from Pozharskiy et al. (2022), as indicated in Table 1.

** According corresponding publications, see references in Table 3.

*** Fragment sizes were not reported by Lanfermeijer, Warmink & Hille (2005). The genotypes were accessed based on the reference gel image from the referenced article.

## Discussion

The results of this study reflect the history and current state of tomato breeding in Kazakhstan. Since the collapse of the Soviet Union in 1991, the development of vegetable breeding and seed production has remained stagnant in independent Kazakhstan due to a shortage of funding and highly qualified experts (Amirov, 2012). The results of the present study have revealed a low genetic diversity of local tomato varieties and their similarity to foreign cultivars. The content of the studied collection of varieties, as well as the list of approved cultivars (The Ministry of Agriculture of the Republic of Kazakhstan, 2009), show the predominant presence of tomato varieties of Russian origin. Such dependence on Russian seed material, which could be traced back to the Soviet period, not only makes local horticulture more vulnerable to political and economic factors, but also decreases the diversity of the genetic resources available for cultivation.

The set of SSR markers used in this study showed limited information content when applied to the considered collection of tomato varieties. According to Botstein et al. (1980), PIC values above 0.5 indicate high information content of a codominant marker, values between 0.25 and 0.5—moderate information content, and values below 0.25—low information content. Of 13 markers used, only two were highly informative, three were moderately informative, four had PIC below 0.25, and four were monomorphic. Consequently, the genetic structure revealed by the Bayesian analysis was weak and provided little information on the possibly classification of the local varieties. Thus, to obtain a molecular genetic basis for tomato breeding in Kazakhstan, further studies are required, following two conditions: (a) a sufficient number of markers covering most parts of the tomato genome; and (b) a wider range of available tomato germplasm from throughout the world, or available data on their diversity and compatibility with used marker sets.

A set of SCAR and CAPS markers of resistance to five diseases revealed a low abundance of corresponding resistance factors not only in the local cultivars, but also in all those studied here. The most common marker, At2, associated with resistance locus *I* against *F. oxysporum,* had an equal proportion of resistant and susceptible variants across varieties; approximately the same ratio, 8:7, was observed in the group of local cultivars. However, this marker displayed no strong genotype distribution pattern in relation to the SSR data. Another *F. oxysporum* resistance marker, Z1063 (locus *I-2*), had an allele associated with resistance in one local cultivar, ‘Meruert’. Based on the specificity of the associated resistance loci to *Fol* races (Chitwood-Brown et al., 2021), resistance to race *Fol-1* is more common than *Fol-2*; further studies should also test resistance factors against *Fol-3*. Four local cultivars had a resistant genotype in the Ph3-gsm marker to *P. infestans,* and no local varieties had resistance markers against the three considered viruses. These results indicate that no systematic approaches have been developed thus far to work with resistance factors in breeding; the observed markers appeared occasionally and without a strong relation to the overall genetic structure.

Despite the role of the former Research Institute of Potato and Vegetable Breeding, in general, the development of tomato breeding in Kazakhstan has been led in a poorly organized and sporadic manner. Because of the losses of information resulting from outdated infrastructures and insufficient funding since the early years of the country’s independence, the origin and subsequent selection of local tomato varieties cannot be traced. The re-establishment of tomato selection in the country at the contemporary level will require joined efforts from the government, farming businesses, and research institutions, including the utilization of modern methods of molecular genetics.

The obtained results demonstrate that further studies with expanded sets of markers and varieties are required, as the data obtained in this work provide limited information. The extension of knowledge about tomato genetics is a crucial aspect of the development of tomato breeding in the country, and particular attention should be paid to the evaluation of a wider range of markers associated with resistance to various diseases and other biotic and abiotic stress factors, supplementing experimental tests. The development of new resistant varieties and their introduction for wide-scale commercial usage will increase the sustainability of the tomato market in Kazakhstan and, thus, help strengthen food safety in the republic. Marker-assisted selection should therefore play a key role in breeding to achieve this goal.

## Conclusions

The results of this study demonstrated the low diversity and weak genetic structure of tomato varieties bred and used in Kazakhstan. The set of 13 SSR markers tested has shown limited applicability for studying the genetic structure of local tomato varieties. The local varieties have shown a low abundance of genetic markers associated with resistance to *Phytophthora infestans* and *Fusarium oxysporum,* and the absence of resistance markers against ToMV, TSMV and TYCWV. The limitations of the obtained results imply the need for further studies employing a wider range of markers and involving more diverse tomato genotypes, which are important for the future development of tomato breeding in Kazakhstan.

##  Supplemental Information

10.7717/peerj.15683/supp-1 Supplemental Information 1 PCR protocols used in the study

10.7717/peerj.15683/supp-2 Supplemental Information 2 SSR genotyping data for tomato varieties The allele size of 13 SSR markers.

10.7717/peerj.15683/supp-3 Supplemental Information 3 Genotyping data for resistance markers of tomato

## Additional Information and Declarations

### Data Availability

The following information was supplied regarding data availability: The raw data is available in the Supplemental Files.
