# Supplementary material for: Genetic diversity of the breeding collection of tomato varieties in Kazakhstan assessed using SSR, SCAR and CAPS markers
Source: PeerJ. 2023 Jul 17;11:e15683. doi: 10.7717/peerj.15683 (PMC10358335; doi:10.7717/peerj.15683)
Supplement: Supplemental Information 1 [file peerj-11-15683-s001.doc]

**Aditional file 1**

*Pozharskiy et al.*

**Genetic diversity of the breeding collection of tomato varieties in Kazakhstan assessed using SSR, SCAR and CAPS markers**

**PCR protocols**

PCR mix:

10x DreamTaq™ Buffer: 2 μl

10 mM dNTP mix: 0.4 μl

10 μM Forward primer: 0.4 μl

10μM Reverse primer: 0.4 μl

5 U/μl DreamTaq™ DNA polymerase: 0.2 μl

Ultra pure water: to 20 μl

Program:

1. LE20592, LE21085, LELE25, LELEUZIP, LEMDDNA, LEPRP4, LESODB, LEATRACAb, LPHSF24, LECHSOD, LEMDDNb (Smulders et al., 1997)

94°C 3 min

(94°C 45 s;

55°C 45 s;

72°C 1 min) – 30 cycles

72°C 3 min

2. TMS63, TMS58 (Areshchenkova & Ganal, 2002)

94°C 3 min

(94°C 1 min;

60°C 1 min;

72°C 2 min) – 45 cycles

72°C 10 min

3. Ph3.gsm (Wang et al., 2016), TG328 (Robbins et al., 2010)

94°C 5 min

(94°C 30 s;

55°C 30 s;

72°C 30 s) – 35 cycles

72°C 10 min

4. At2, Z1063 (Arens et al., 2010)

10x DreamTaq™ Buffer: 2 μl

10 mM dNTP mix: 0.4 μl

10 μM Forward primer: 0.4 μl (At2), 0.6 μl (Z1063)

10μM Reverse primer: 0.4 μl (At2), 0.6 μl (Z1063)

10 μM Control forward primer: 0.4 μl (At2), 0.2 μl (Z1063)

10μM Control reverse primer: 0.4 μl (At2), 0.2 μl (Z1063)

5 U/μl DreamTaq™ DNA polymerase: 0.2 μl

Ultra pure water: to 20 μl

Program:

94°C 5 min

(94°C 30 s;

55°C 30 s;

72°C 30 s) – 35 cycles

72°C 10 min

5. PrRuG086-151 (Lanfermeijer, Warmink & Hille, 2005)

94°C 5 min

(94°C 15 s;

55°C 45 s;

72°C 90 s) – 30 cycles

72°C 5 min

6. Sw-5–2 (Dianese et al., 2010)

94°C 2 min

(94°C 30 s;

50°C 1 min;

72°C 30 s) – 30 cycles

72°C 5 min

7. NCSw-003, NCSw-012, NCSw-007, NCSw-011 (Panthee & Ibrahem, 2013)

94°C 3 min

(94°C 18 s;

50°C 1 min;

72°C 18 s) – 35 cycles

72°C 8 min

8. Ty2-UpInDel (*T*_ann._=57.3°C), Ty3-InDel/SNP9 (*T*_ann._=60°C), Ty3-SNP17 (*T*_ann._=58.5°C) (Kim et al., 2020)

94°C 3 min

(94°C 30 s;

*T*_ann_.  30 s;

72°C 45 s) – 40 cycles

72°C 7 min
